# Effects of Thermal Conditioning on Changes in Hepatic and Muscular Tissue Associated With Reduced Heat Production and Body Temperature in Young Chickens

**DOI:** 10.3389/fvets.2020.610319

**Published:** 2021-01-18

**Authors:** Yoshimitsu Ouchi, Vishwajit S. Chowdhury, John F. Cockrem, Takashi Bungo

**Affiliations:** ^1^Laboratory of Animal Behavior and Physiology, Graduate School of Integrated Sciences for Life, Hiroshima University, Higashihiroshima, Japan; ^2^Division for Experimental Natural Science, Faculty of Arts and Science, Kyushu University, Fukuoka, Japan; ^3^School of Veterinary Science, Massey University, Palmerston North, New Zealand

**Keywords:** poultry, thermal conditioning, ASS, ASL, arginosuccinate acid

## Abstract

Effects of increased summer temperatures on poultry production are becoming more pronounced due to global warming, so it is important to consider approaches that might reduce heat stress in chickens. Thermal conditioning in chickens in the neonatal period can improve thermotolerance and reduce body temperature increases when birds are exposed to high ambient temperature later in life. The objective of this study was to investigate physiological and molecular changes associated with heat production and hence body temperature regulation under high ambient temperatures in thermally conditioned chicks. Three-day-old broiler chicks (Chunky) were thermally conditioned by exposure to a high ambient temperature (40°C) for 12 h while control chicks were kept at 30°C. Four days after the treatment, both groups were exposed to 40°C for 15 or 90 min. The increase in rectal temperature during 90 min of exposure to a high ambient temperature was less in thermally conditioned than control chicks. At 15-min of re-exposure treatment, gene expression for uncoupling protein and carnitine palmitoyletransferase 1, key molecules in thermogenesis and fatty acid oxidation, were significantly higher in pectoral muscle of control chicks but not conditioned chicks. Hepatic argininosuccinate synthase (ASS) decreased and hepatic argininosuccinate lyase (ASL) increased after reexposure to a high temperature. The concentrations of hepatic arginosuccinic acid, and ASS and ASL expression, were upregulated in conditioned chicks compared with the control chicks, indicating activity of the urea cycle could be enhanced to trap more energy to reduce heat production in conditioned chicks. These results suggest thermal conditioning can reduce the increase in heat production in muscles of chickens that occurs in high ambient temperatures to promote sensible heat loss. Conditioning may also promote energy trapping process in the liver by altering the heat production system, resulting in an alleviation of the excessive rise of body temperature.

## Introduction

The increased magnitude and duration of elevated summer temperatures due to the advance of global warming is a pressing challenge that should be addressed from not only the viewpoint of adverse effects on health but also from considerations of food production. When animals experience high air temperatures it is commonly said that they are experiencing heat stress, but this term is rarely defined. The definition used here is that heat stress is a situation when an animal's thermoregulatory mechanisms are unable to prevent body temperature from increasing above the thermoneutral zone. Livestock can show loss of appetite and reduced rates of weight gain when they experience heat stress in summer and in extreme cases animals can die due to heat stress (1–3). If the incidence of heat stress in animals continues to increase at the current rate then adverse effects due to summer heat will start to occur in areas that are currently cold, leading to decreases in livestock productivity in summer in these areas (4).

Chickens are the most consumed edible meat species in the world, with 130.5 million tons of chicken meat produced annually (5). On the other hand, chickens are susceptible to summer heat because poultry do not have sweat glands and have limited capability for sensible heat loss. In addition, broilers have lower heat tolerances than layers due to high growth performance and large muscle tissue mass as a result of selective breeding for these characteristics (6, 7). Furthermore, male broilers are more susceptible to heat than females (8). Increasing temperatures due to global warming are having a major negative impact on the poultry industry (9, 10). Whilst a variety of approaches such as improvements in rearing systems and the supply of feed additives that mitigate heat stress have been applied, effective strategies are needed to reduce the effects of elevated temperatures on chickens. The relationship between genetic polymorphism and thermotolerance has been reported (11), and the development of thermotolerance breeds based on that report is one of the methods to respond heat stress. However, breeding takes a long time and new strategies are needed to reduce effects of heat stress on chickens.

Thermal conditioning is a technique that can increase the tolerance of chickens to high temperatures. The treatment involves exposure of chicks to a high ambient temperature early in life (12–14). The treatment can increase thermotolerance in chickens and this approach might be an effective method to mitigate heat stress in chickens. It has been reported that the improvement of thermotolerance by thermal conditioning is due to the modification of the central thermoregulatory system (15). Gene expression in this system can be regulated by thermal conditioning as a consequence of the high plasticity of central nervous system neurons in young animals (16, 17). It has been suggested that adaptation to high temperature environments might be due to epigenetic modifications such as changes to brain derived neurotropic factor (BDNF) and corticotropin-releasing hormone (CRH) genes (18–20). On the other hand, the effects of thermal conditioning on peripheral organs are not clear. Yahav and McMurtry (14) reported that thermal conditioning at 3 days of age in broiler chickens affected weight gain. Moreover, muscle and peripheral organs actually have the function of generating body heat. Therefore, it is suggested that thermal conditioning may affect muscle and peripheral organs in chickens.

Body temperature can be regulated by the physiological process of non-shivering thermogenesis and by metabolic processes. In non-shivering thermogenesis, skeletal muscle is an important organ in birds (21). The development of muscle fibers is associated with actin and myosin, which are the major proteins in myofibrils (22). Therefore, examination of actin and myosin could provide important information on muscle protein activity related to body temperature. Furthermore, it has been suggested that mitochondrial uncoupling protein (UCP) acts as a mitochondrial anion carrier in thermogenesis (23). Additionally, carnitine-palmitoyltransferase (CPT)-1, an enzyme for mitochondrial fatty acid transport (24), is involved in thermogenesis in birds (25). Chowdhury et al. (26) reported that plasma L-citrulline (L-Cit) concentrations declined in heat-exposed chicks and oral administration of L-Cit lead to thermotolerance (27). Therefore, it could be predicted that the L-Cit metabolic pathway could be modulated by thermal conditioning. L-Cit is converted to argininosuccinic acid by argininosuccinate synthease (ASS) and argininosuccinic acid is further metabolized to arginine by argininosuccinate lyase (ASL). Arginine is then converted to L-Cit or ornithine. Examination of the enzymes of the urea cycle could provide information on metabolic modulation by thermal conditioning.

The aim of this study was to clarify the effect of thermal conditioning at young ages on heat production and heat dissipation in chickens. We investigated the effects of thermal conditioning on heat dissipation behavior and thermogenesis related molecules in the major heat-producing organs, the liver and muscle.

## Materials and Methods

The handling of birds was performed in accordance with the regulations of the Animal Experiment Committee of Hiroshima University (Authorization No. C19-15) and complied with Law No. 105 and Notification No. 6 of the Japanese government.

### Animals

Day-old male broiler chicks (Chunky, *n* = 72) were obtained from local hatcheries (Fukuda Hatchery Co., Ltd., Okayama, Japan). Chicks were maintained in a room at a temperature of 30°C with 24-h lighting. The chicks were kept in wooden cages with a wire-mesh floor (18 × 25 × 20 cm) at a population density of three chicks per cage during the pre-experimental period. They were given free access to a commercial starter diet (metabolizable energy >12.6 MJ/kg and crude protein >23%; Nichiwa Sangyo Co. Ltd., Kobe, Japan) and water until the end of experiments. At 2 days of age birds were distributed into groups based on their body weight so that the average body weight was as uniform as possible for each treatment. The chicks were kept singly in wooden cages during experimental periods after treatments.

### Experiment Design

Thermal conditioning treatments consisted of moving birds at 3 days of age and exposing them to high ambient temperature for 12 h in a commercial incubator (Type P-008B, Showa Furanki, Saitama, Japan). The temperature and relative humidity of thermal conditioning treatment were 40 ± 0.5°C and 65 ± 5%, respectively. The thermal conditioning processed was based on previous studies (14, 28, 29). Birds had free access to food and water during the thermal conditioning treatments. Non-thermal conditioning treated chicks were kept under the thermoneutral zone.

### Experiment 1: Effect of Thermal Conditioning on Growth and Muscular Gene Expressions of Myosin and Actin

**Figure 1:**
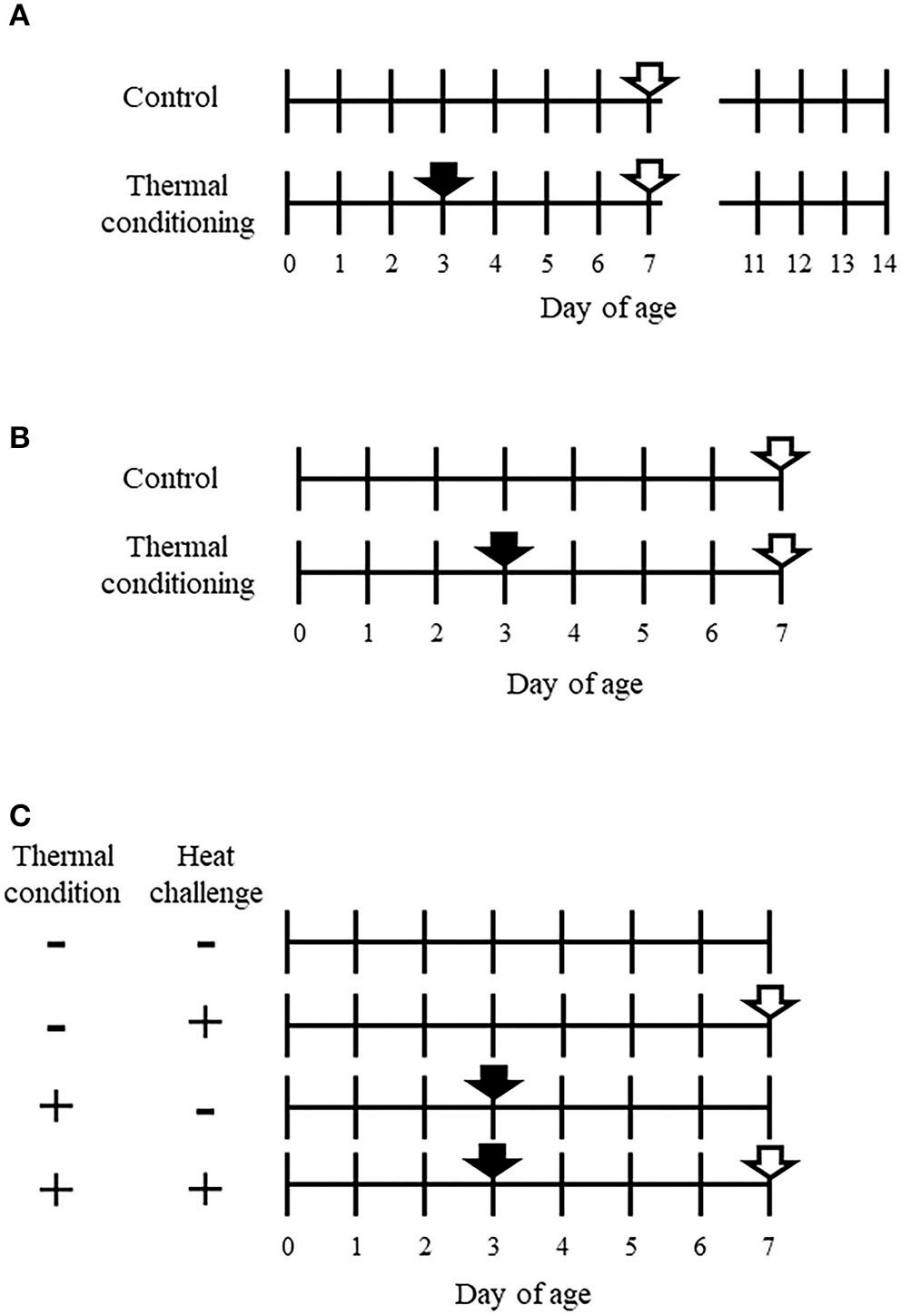
Experimental designs of this study. **(A)** Design of experiment 1. A closed arrow indicates the thermal conditioning treatment. Closed arrows indicate time points of sampling for collecting pectoral muscle. The total number of chicks was 32, with 16 chicks per group. The Pectoral muscle tissue was collected from half of the chicks in each group at 7 days old. Other Body weight was measured daily from 2 to 14 days of age in the other half of the chicks in each group. **(B)** Design of experiment 2. A closed arrow shows thermal conditioning and open arrows show heat challenge. The number of chicks was eight per group. **(C)** Design of experiment 3. + indicates treated and – indicates non-treated. Closed arrows show thermal conditioning treatment and open arrows indicate heat challenge. The number of chicks was eight per group. Four chicks were used for metabolome analysis in each group.

The experimental design is shown in Figure 1A. The total number of chicks used for experiment 1 was 32. Chicks were divided into a control group and a thermal conditioning group (*n* = 16 per group). At 7 days-old, half of the chicks in each group were selected in consideration of the average body weight of each group and euthanized. Pectoral muscle tissues were collected for measurement gene expression of myosin and actin. These tissue samples were stored at −80°C until analysis. The other chicks of both groups were weighed daily from 2 days of age to 14 days of age.

### Experiment 2: Effect of Heat Challenge on Rectal Temperature and Thermoregulation Behavior in Thermally Conditioned Chicks

The experimental design is shown in Figure 1B. Chicks were divided into a control group and a thermal conditioning group (*n* = 8 per group, total chicks *n* = 16). At 7 days-old, both groups of chicks were challenged to high ambient temperature for 90 min. The temperature and relative humidity were 40 ± 0.5°C and 66 ± 5%. This heat challenge was designed with reference to Chowdhury et al. (30). The treatment was carried out in a commercial incubator (Type P-008B, Showa Furanki, Saitama, Japan). Rectal temperature was measured every 30 min (0, 30, 60, 90 min) during the heat challenge. The times when thermoregulation behaviors (panting and wing-drooping) began in chicks were visually observed and recorded as described elsewhere (15, 29).

### Experiment 3: Effect of Thermal Conditioning on Hepatic and Muscular Parameters After Heat Challenge

The experimental design is shown in Figure 1C. Birds were divided into four groups (*n* = 8 per group, total *n* = 24), (1) no treatment (control group), (2) exposure to high ambient temperature (heat challenge) for 15 min at 7-days-old without thermal conditioning treatment, (3) thermal conditioning treatment, (4) thermal conditioning treatment and heat challenge. This heat challenge was designed based on previous study (15, 29). The temperature and relative humidity of heat challenge were 40 ± 0.5°C and 66 ± 5%. After heat challenge, all groups of chicks were decapitated after anesthesia and hepatic and pectoral muscle tissues were collected and stored at −80°C until RNA isolation were performed. The collected hepatic tissues for metabolome analysis (*n* = 4 per group) were also stored as tissues for gene expression analysis.

### Measurements of Hepatic and Muscle Concentrations of Metabolites Related to Energy Metabolism

Metabolome measurements were carried out through a facility service at Human Metabolome Technologies Inc., Tsuruoka, Japan. Approximately 50 mg of frozen hepatic tissue was plunged into 1,500 μL of 50% acetonitrile/Milli-Q water containing internal standards (H3304-1002, Human Metabolome Technologies, Inc., Tsuruoka, Japan) at 0°C in order to inactivate enzymes. L-Methionine sulfone was used for the internal standards of cation mode and D-camphor-10-sulfonic acid was used for the internal standards of anion mode. The tissue was homogenized thrice at 1,500 rpm for 120 s using a tissue homogenizer (Micro Smash MS100R, Tomy Digital Biology Co., Ltd., Tokyo, Japan) and then the homogenate was centrifuged at 2,300 × g and 4°C for 5 min. Subsequently, 800 μL of upper aqueous layer was centrifugally filtered through a Millipore 5-kDa cutoff filter at 9,100 × g and 4°C for 120 min to remove proteins. The filtrate was centrifugally concentrated and re-suspended in 50 μL of Milli-Q water for CE-TOFMS analysis. CE-TOFMS measurements were carried out using an Agilent CE Capillary Electrophoresis System equipped with an Agilent 6210 Time of Flight mass spectrometer, Agilent 1100 isocratic HPLC pump, Agilent G1603A CE-MS adapter kit, and Agilent G1607A CE-ESI-MS sprayer kit (Agilent Technologies, Waldbronn, Germany). The systems were controlled by Agilent G2201AA ChemStation software version B.03.01 for CE (Agilent Technologies, Waldbronn, Germany). The metabolites were analyzed by using a fused silica capillary (50 μm i.d. × 80 cm total length), with commercial electrophoresis buffer (Solution ID: H3301-1001 for cation analysis and H3302-1021 for anion analysis, Human Metabolome Technologies) as the electrolyte. The sample was injected at a pressure of 50 mbar for 10 s (~10 nL) in cation analysis and 25 s (~25 nL) in anion analysis. The spectrometer was scanned from m/z 50 to 1,000. Other conditions were as in the described previously (31–33). Peaks were extracted using automatic integration software MasterHands (Keio University, Tsuruoka, Japan) in order to obtain peak information including m/z, migration time for CE-TOFMS measurement (MT) or retention time for LC-TOFMS measurement (RT), and peak area (34). Signal peaks corresponding to isotopomers, adduct ions, and other product ions of known metabolites were excluded, and remaining peaks were annotated with putative metabolites from the HMT metabolite database based on their MTs/RTs and m/z values determined by TOFMS. The tolerance range for the peak annotation was configured at ±0.5 min for MT and ±10 ppm for m/z. In addition, peak areas were normalized against those of the internal standards and then the resultant relative area values were further normalized by sample amount. Hierarchical cluster analysis (HCA) and principal component analysis (PCA) were performed by our proprietary software, PeakStat and SampleStat, respectively. Detected metabolites were plotted on metabolic pathway maps using VANTED (Visualization and Analysis of Networks containing Experimental Data) software (35).

### Measurements of Hepatic and Muscle Gene Expression

RNA of hepatic or muscular tissue was isolated from the dissected tissue using Trizol reagent (Invitrogen, CA, USA) according to the manufacturer′s instructions. To rule out the possibility that PCR products would result from the amplification of genomic DNA contaminating the RNA sample, RNA samples were treated with DNase I using the DNA-free kit (Ambion, Austin, USA). Total RNA (500 ng) was reverse transcribed at 42°C for 15 min in 10 μl of 1 × Prime Script RT Enzyme Mix I (Takara, Shiga, Japan). The reaction product was subjected to real-time PCR performed according to the user instructions for the Light Cycler system (Roche Life Science, Basel, Switzerland). In brief, following a denaturation step at 95°C for 600 sec, PCR was carried out with a thermal protocol consisting of 95°C for 10 s and 60°C for 20 s in a 20 μl buffer containing 2 × FastStart Essential DNA Green Master (Roche Life Science) and 0.2 μM of each primer. Primers used for real-time PCR are shown in Table 1. The genes analyzed were avian uncoupling protein (avUCP), carnitine palmitoyltransferase-1 (CPT1), actin and myosin in pectoral muscle. The genes analyzed in hepatic tissue were argininosuccinate synthase (ASS) and argininosuccinate lyase (ASL). To normalize the data, ΔC_T_ was calculated for each sample by subtracting C_T_ of RPS17 from C_T_ of the gene of interest. For relative quantitation, ΔC_T_ for the defined control group was subtracted from the ΔC_T_ of each experimental sample to generate ΔΔC_T_. The ΔΔC_T_ was then used to calculate the approximate fold difference, 2^−ΔΔ*CT*^. The results were expressed as the relative gene expression.

**Table 1:** Primer sequences for real-time PCR in this study.

**PCR assay**	**Forward (5^**′**^ → 3^**′**^)**	**Reverse (5^**′**^ → 3^**′**^)**	**Accession No**.
RPS17	AAGCTGCAGGAGGAGGAGAGG	GGTTGGACAGGCTGCCGAAGT	NM_204217
ASS	AGAACCTCATGCACATCAGC	TTGGGTTGCAGGTCTTTGTG	NM_001013395.1
ASL	AAAACGTCGTCCGAAATGGC	ATCTCCATGATGGGATCTGTGC	NM_205501.2
avUCP	ACCAACACGGTGGAGTACC	TGGAGGCGAAGCTCATC	NM_204107
CPT-1	TCAGACACCACAGCAACACA	ATCAGCCACAGGTCCAAATC	AY675193
Myosin	TTGCAGAGTCGCAAGTCAAC	TTCACTTTGCACCHCTTGAG	V00430.1
Actin	TTGTCCACCGCAAATGCTTC	AAAGCCATGCCAATCTCGTC	NM_205518.1

*RPS17, ribosomal protein S17; ASS, argininosuccinate synthase; ASL, argininosuccinate lyase; avUCP, avian uncoupling protein; CPT-1, carnitine palmitoyltransferase-1; Myosin, myosin heavy chain; Actin, actin beta*.

### Statistical Analysis

The data were analyzed using the commercially available package, StatView (Version 5, SAS Institute, Cary, USA, 1998). At first, all date were analyzed by test of rejection on Smirnoff-Grubbs. Body weight and rectal temperature data were analyzed by repeated measures two-way ANOVA (Generalized Linear Model). Two-way factorial ANOVAs (Generalized Linear Model) were used to compare treatments for hepatic concentrations of argininosuccinic acid and for hepatic and muscle gene expression. When significant interactions were found in ANOVA analyses *post-hoc* tests were performed using the Tukey–Kramer test to compare treatments. *T*-tests were used to compare muscle gene expression between control and thermal conditioning groups in the first experiment. Differences were declared significant at *P* < 0.05, and a value of *P* ≤ 0.10 was considered to reflect a trend toward significance.

## Results

### Experiment 1: Effect of Thermal Conditioning on Body Weight Gain and Gene Expression in Pectoral Muscle

**Figure 2:**
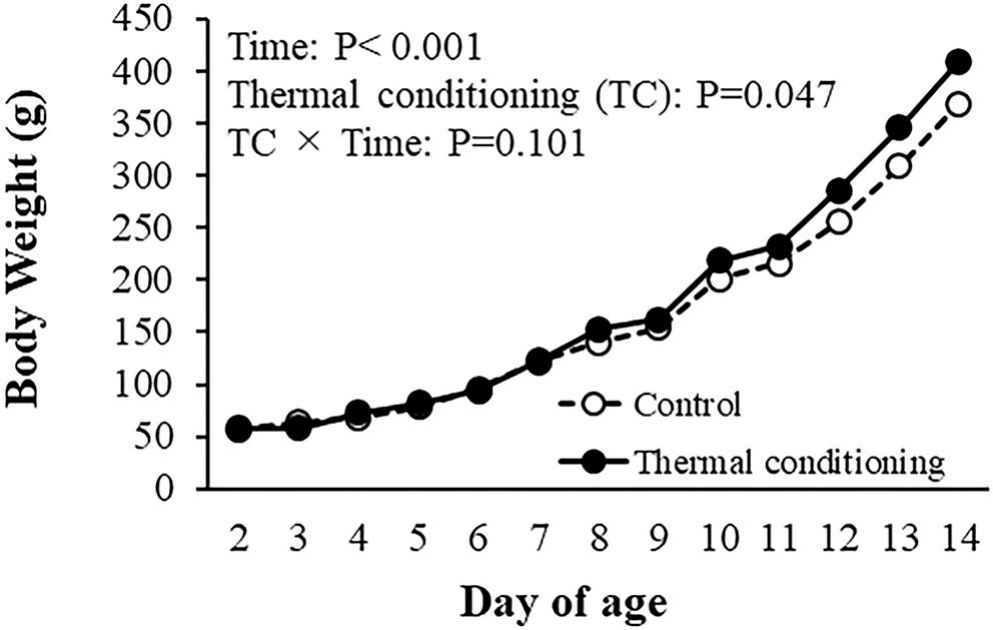
Effect of thermal conditioning on body weight in chicks. Open circles indicate the control group (no-thermal conditioning). Closed circles indicate the thermal conditioning (TC) group. The number of chickens in each group was *n* = 8.

The effects of thermal conditioning on body weight in chicks are shown in Figure 2. There were significant effects of age (*P* < 0.001) and treatment (*P* = 0.047).

**Figure 3:**
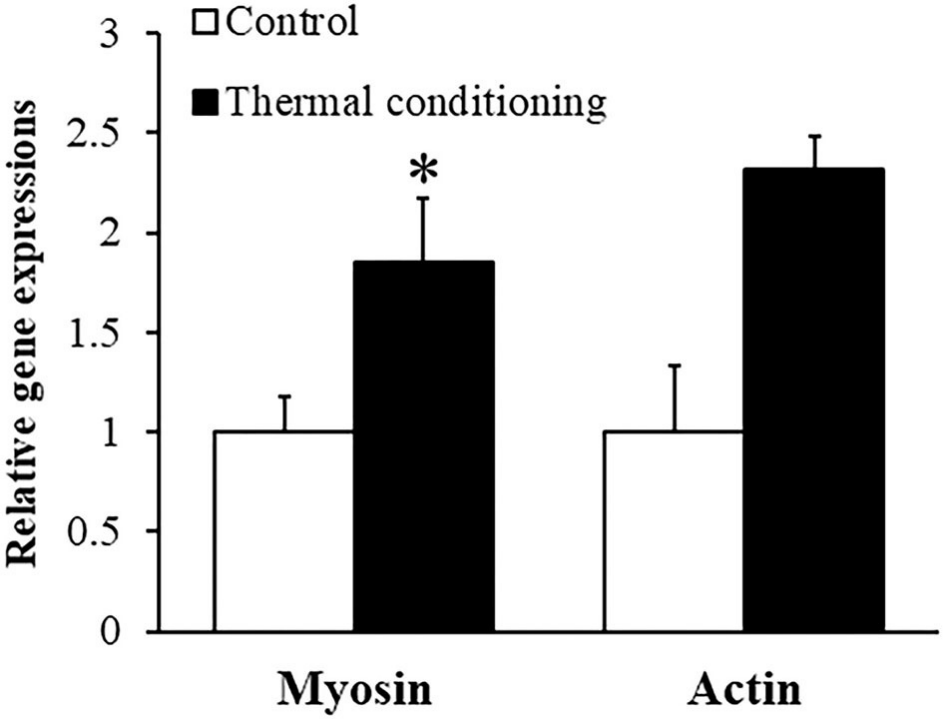
Effect of thermal conditioning on gene expression in pectoral muscle in chicks. Asterisks refer to the level of statistical significance (*P < 0.05). Data were expressed as means ± SEM. The number of chickens in each group was *n* = 7–8.

Figure 3 shows the effect of thermal conditioning on myosin and action gene expressions in pectoral muscle of chicks. Myosin and actin expression levels were both higher in thermal conditioned chicks than in control chicks (*P* = 0.041 and *P* = 0.034).

### Experiment 2: Effect of Heat Challenge on Rectal Temperature and Thermoregulation Behavior in Thermal Conditioned Chicks

**Figure 4:**
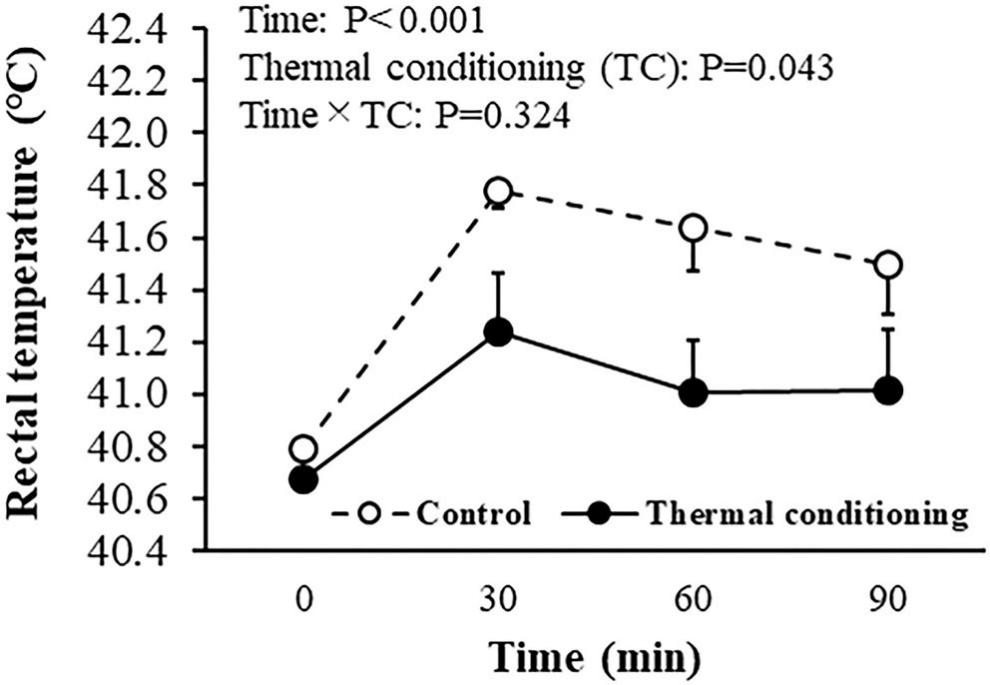
Effect of thermal conditioning on rectal temperature during heat challenge in chicks. Open circles indicate the control group (no-thermal conditioning). Closed circles indicate the thermal conditioning group. The number of chickens in each group was *n* = 8.

Figure 4 shows the effect of thermal conditioning on rectal temperature during a 90 min heat challenge at 7 days of age in chicks. Rectal temperature increased during the heat challenge (*P* < 0.001) in control chicks and in chicks that had experienced thermal conditioning. The elevation of rectal temperature was lower in thermally conditioned chicks (*P* = 0.043). Initial times of wing drooping were not affected by thermal conditioning (control: 628 ± 157.2 sec, treatment: 637 ± 197.9 s, *P* = 0.814). Similarly, thermal conditioning did not affect the initial time of panting (control: 845 ± 149.2 sec, treatment: 705 ± 172.4 sec, *P* = 0.769).

### Experiment 3: Effect of Thermal Conditioning on Hepatic Tissue and Pectoral Muscle After Heat Challenge

**Figure 5:**
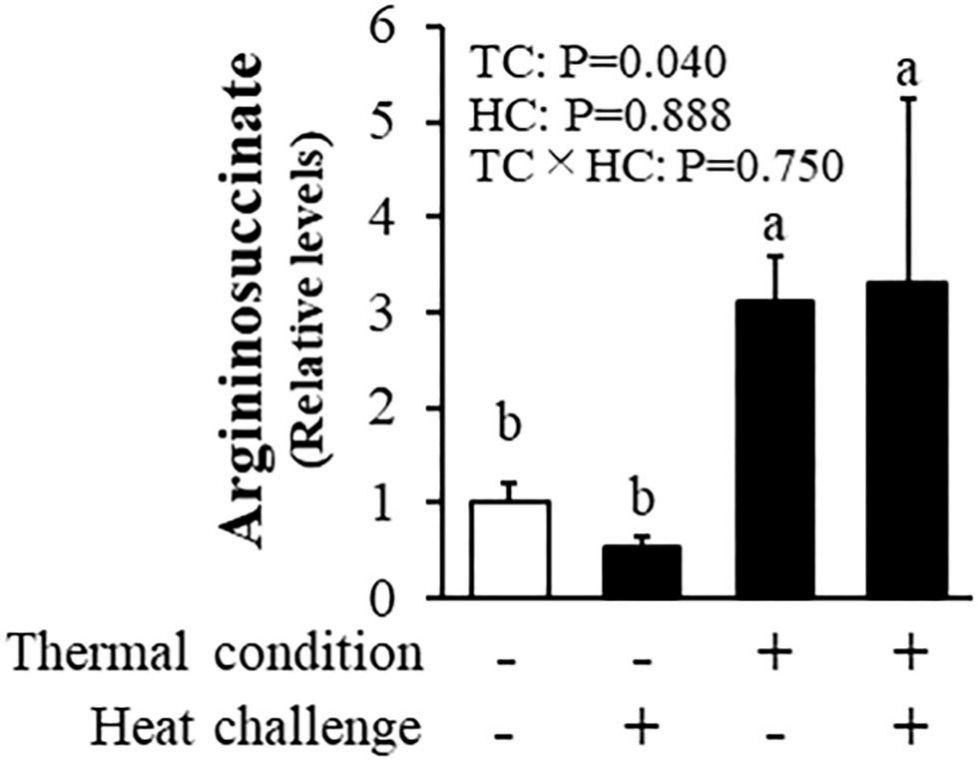
Effect of thermal conditioning on levels of hepatic argininosuccinic acid in chicks. TC, thermal conditioning; HC, heat challenge. The number of chickens in each group was *n* = 4. +: treatment, –: non-treatment. There is a significant difference between different characters.

As a result of metabolome analysis, 302 metabolites detected by CE-TOFMS. Figure 5 shows levels of hepatic argininosuccinic acid measured by metabolomic analysis. There was a significant effect of thermal conditioning, no effect of heat challenge and no significant interaction, with levels of hepatic argininosuccinic acid higher in conditioned than control chicks (*P* = 0.040). There were no effects of thermal conditioning on levels of other measured metabolites in urea-cycle (data not shown).

**Figure 6:**
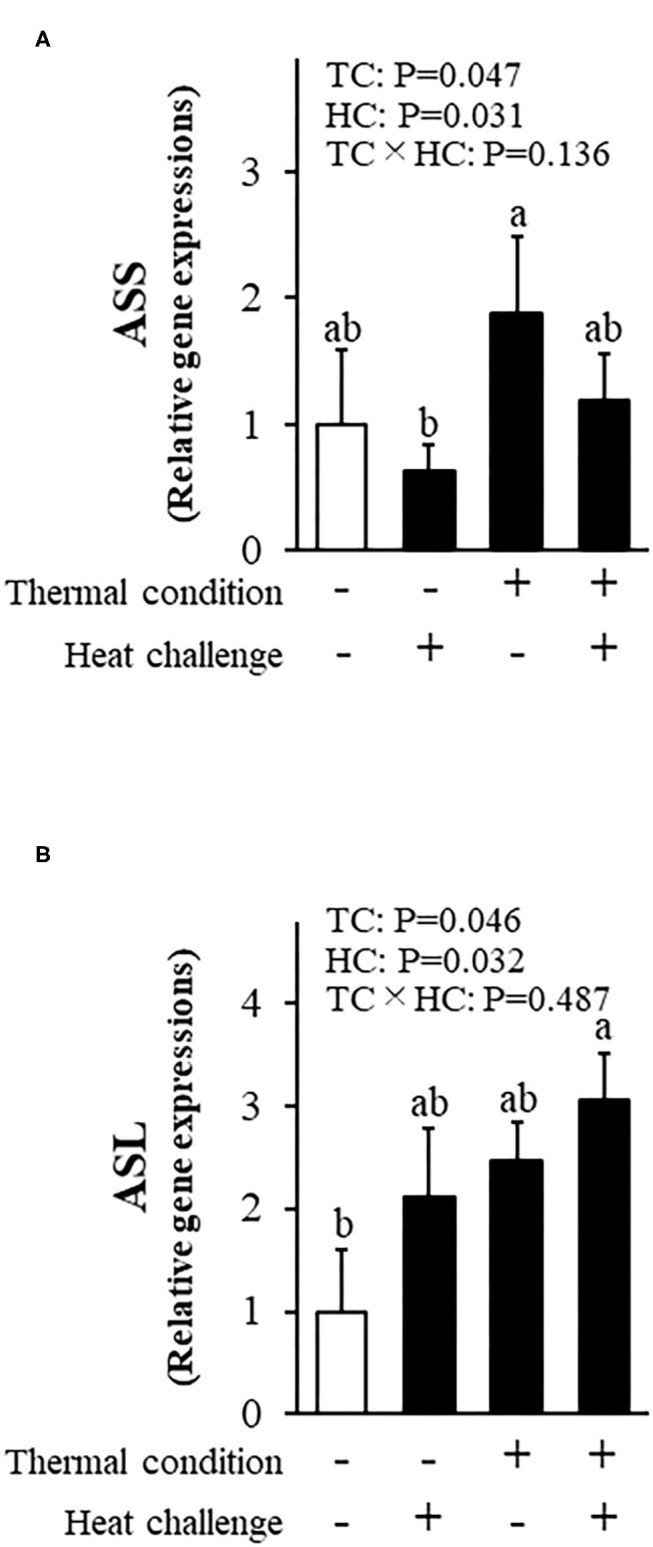
Effect of thermal conditioning on hepatic gene expressions in chicks. **(A)** argininosuccinate synthase (ASS), **(B)** argininosuccinate lyase (ASL). TC, thermal condition; HC, heat challenge. Data were expressed as means ± SEM. The number of chickens in each group was *n* = 8. +: treatment, –: non-treatment. There is a significant difference between different characters.

Figure 6 shows the effect of thermal conditioning on hepatic expression of genes related to argininosuccinic acid in chicks after heat challenges. There were significant effects of thermal condition and heat challenge on argininosuccinate synthase expression, with no significant interaction between thermal condition and heat challenge. The expression of argininosuccinate lyase was affected by thermal conditioning and by heat challenge, with no significant interaction.

**Figure 7:**
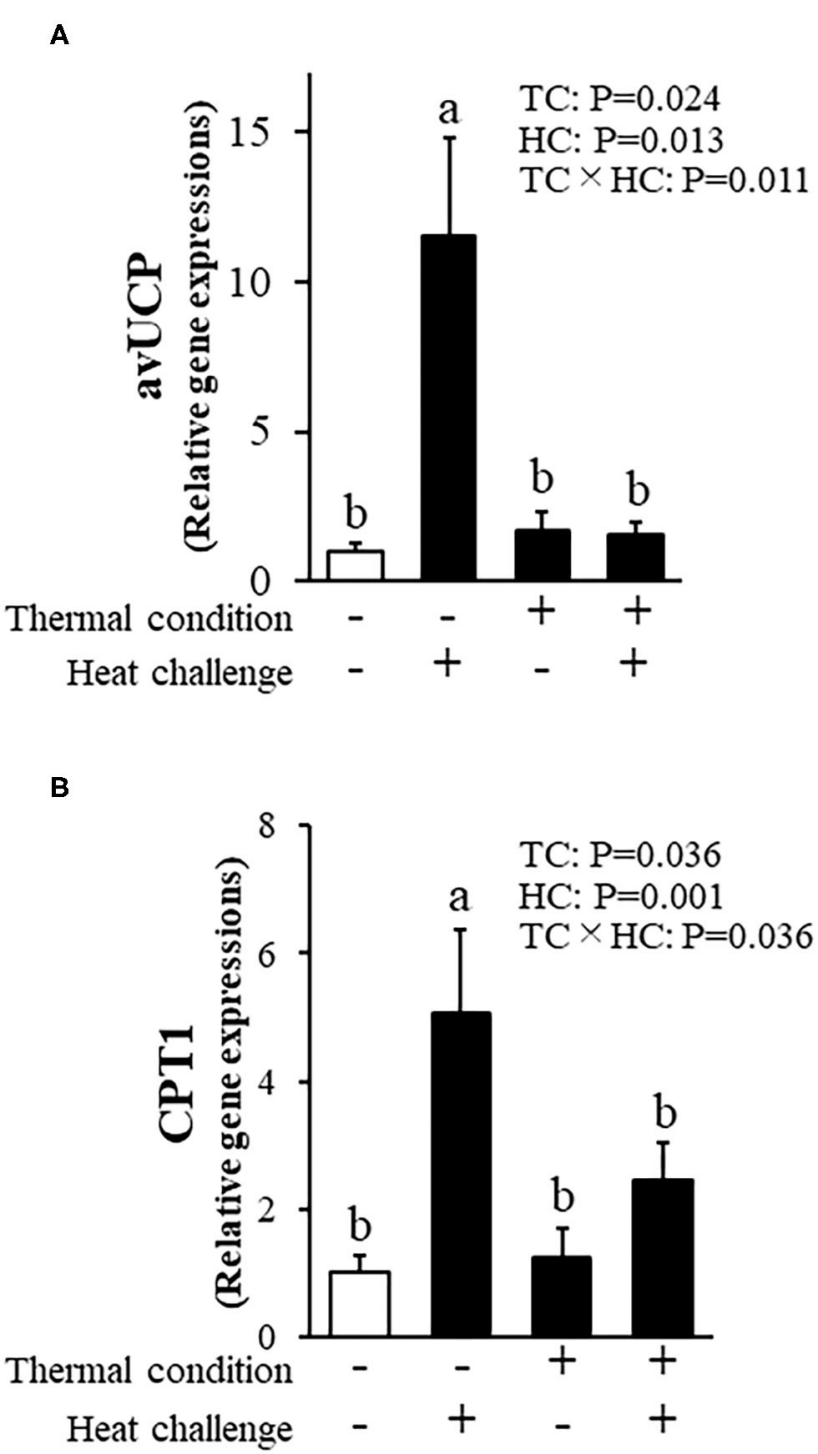
Effect of thermal conditioning on gene expression in the pectoral muscle of chicks. **(A)** Avian uncoupling protein (av-UCP), **(B)** carnitine palmitoyltransferase-1 (CPT1). TC, thermal condition; HC, heat challenge. Data were expressed as means ± SEM. Means with different letters are significantly different at *P* < 0.05. The number of chickens in each group was n = 7–8. +: treatment, –: non-treatment. There is a significant difference between different characters.

Figure 7 shows avian uncoupling protein (av-UCP) and carnitine palmitoyltransferase-1 (CPT1) expression in pectoral muscle. There were significant treatment effects and interactions for av-UCP expression. Av-UCP expression after heat challenge in chicks was increased in control chicks (*P* = 0.011) and not in thermally conditioned chicks. Heat challenge also elevated CPT1 expression in control but not thermally conditioned chicks (*P* = 0.036).

## Discussion

There are many reports of effects of early thermal manipulation on growth performance in chickens, with variable results from different studies (14, 28). Our results agreed with prior studies that showed early thermal conditioning to enhance body weight gain in chickens (14). Myosin and actin are major structural proteins in skeletal muscle. The expression of myosin and actin genes was upregulated in thermally conditioned chicks and Halevy et al. (36) showed that thermal manipulation at an early age significantly improved muscle mass at a later age in chicks. Thermal conditioning appears to cause activation of protein synthesis and pectoral muscle hypertrophy in chickens which leads to improved body weight gain. Heat stress partially induces muscle hypertrophy via heat shock transcription factor [HSF: (37)], and heat stress increases the expression of HSF in the muscle of chickens (38). Additionally, thermal conditioning at 3 days of age enhanced intestinal growth and nutrient intake in chickens (39) and thermal conditioning might contribute to growth via increased development of the intestinal tract and improved efficiency of nutrient absorption.

The increase in rectal temperatures during a heat challenge was lower in thermally conditioned chicks than in control chicks in the current study. Previous studies also showed that thermal conditioning reduced the magnitude of rectal temperature increases in chickens after heat exposure (14, 15, 40). The alleviation of hyperthermia caused by heat exposure could be due to decreased heat production or to increased heat dissipation. In chickens, heat dissipation can occur by sensible heat loss from the body surface such as wing drooping and by latent heat loss via panting, but cannot occur via sweating as sweat glands are absent (37). The initiation of panting and wing drooping were not changed by thermal conditioning in the present study. Thus, thermal conditioning may alter heat production but not heat dissipation behavior in chicks.

It is well-known that there are three major heat production pathways in animals; (1) metabolic heat production, (2) shivering heat production, and (3) non-shivering heat production. Metabolic heat production is always occurring as it is necessary to produce energy by metabolism for the maintenance of life. A reduction of metabolism leads to lower heat production and to lower body temperature in chickens (41). The liver is one of the most important organs for metabolism in animals. In the present study, the level of hepatic argininosuccinic acid was increased by thermal conditioning. Argininosuccinate synthase and argininosuccinate lyase catalyze the formation and breakdown of argininosuccinic acid (42). The expression of genes for both of these enzymes was increased by thermal conditioning. Argininosuccinic acid is an amino acid metabolized in the urea cycle, so there is a possibility that the heat production system associated with the urea cycle may be altered by thermal conditioning. This concept is supported by reports that oral L-citrulline, which is an amino acid synthesized and degraded in urea cycle, induced hypothermia and thermotolerance in broilers (27, 43). Separately, L-arginine in the urea cycle is a substrate for nitric oxide synthases to generate nitric oxide (44). Nitric oxide affects various behaviors including thermoregulatory behavior (45, 46), so it is possible that increased nitric oxide affected body temperature in chicks. However, Chowdhury et al. (27) showed that citrulline-induced hypothermia was not involved in nitric oxide production, so it is unlikely that nitric oxide contributed to the alleviation of body temperature rise that was induced by thermal conditioning.

Non-shivering heat production, a thermogenesis mechanism in animals, is mainly due to uncoupling of oxidative phosphorylation in mitochondria. Uncoupling proteins have important roles in non-shivering heat production (47) and are activated by triiodothyronine (T3) (47, 48). Thermal conditioning in the present study reduced the magnitude of an increase in uncoupling protein gene expression in pectoral muscle that was induced by a heat challenge, implying that heat production in pectoral muscle might be decreased by thermal conditioning. This would be consistent with reports that thermal conditioning reduced plasma T3 levels during heat exposure after growth (13, 39), and the expression of the UCP gene in the pectoral muscle was decreased after two days of thermal conditioning (49). Carnitine palmitoyltransferase-1 (CPT1) is the rate-limiting enzyme in the oxidation of fatty acids (50). We found that an increase of CPT1 gene expression in pectoral muscle after a heat challenge was suppressed in thermally conditioned chicks. These results suggest that mitochondrial fatty acid uptake was suppressed during a heat challenge in thermally conditioned chicks. It has been reported that fatty acids transferred to skeletal muscle enhance UCP gene expression level in mice (51, 52). The increase of UCP gene expression in non-treated chicks could be attributed to the increase of CPT1 expression whilst fatty acid uptake and avUCP expression were suppressed in thermally conditioned chicks during a heat challenge.

The central nervous system is important for the regulation of body temperature in homeotherms including chickens. Thus, it has been suggested that the improvement of thermotolerance associated with early thermal manipulation is due to plastic changes in the brain, especially the hypothalamus. Yossifoff et al. (18) reported that methylation levels of hypothalamic BDNF gene were associated with thermal conditioning-induced heat tolerance. Also, Tanizawa et al. (15) indicated that early thermal conditioning changed thermoregulatory systems in the hypothalamus, resulting in resistance to heat stress. Our results showed that thermotolerance can also be obtained by the use of thermal conditioning to change heat production of peripheral tissues such as liver and muscle. As De Basilio et al. (28) also indicated, thermal conditioning induces metabolic changes in chickens. Methylation in peripheral tissues may be associated with thermal conditioning-induced heat tolerance and this possibility warrants further investigation.

In conclusion, the current results suggest that thermal conditioning treatment can reduce metabolic heat production and non-shivering heat production in chicks when under high ambient temperatures, can activate protein synthesis in pectoral muscle, and can improve body weight gain without increasing heat production associated with weight gain. Furthermore, thermal conditioning is an effective technique to reduce heat stress in young chickens.

## Data Availability Statement

The original contributions presented in the study are included in the article/supplementary materials, further inquiries can be directed to the corresponding author/s.

## Ethics Statement

The animal study was reviewed and approved by regulations of the Animal Experiment Committee of Hiroshima University (Authorization No. C19-15).

## Author Contributions

All authors listed have made a substantial, direct and intellectual contribution to the work, and approved it for publication.

## Conflict of Interest

The authors declare that the research was conducted in the absence of any commercial or financial relationships that could be construed as a potential conflict of interest.
